# A 2-Dose AERAS-402 Regimen Boosts CD8^+^ Polyfunctionality in HIV-Negative, BCG-Vaccinated Recipients

**DOI:** 10.3389/fimmu.2021.673532

**Published:** 2021-06-11

**Authors:** Dhanasekaran Sivakumaran, Gretta Blatner, Rasmus Bakken, David Hokey, Christian Ritz, Synne Jenum, Harleen M. S. Grewal

**Affiliations:** ^1^ Department of Clinical Science, Bergen Integrated Diagnostic Stewardship Cluster, Faculty of Medicine, University of Bergen, Bergen, Norway; ^2^ Department of Microbiology, Haukeland University Hospital, University of Bergen, Bergen, Norway; ^3^ Biomedical Advanced Research and Development Authority (BARDA), Department of Health and Human Services, Washington, DC, United States; ^4^ Aeras Global TB Vaccine Foundation, Rockville, MD, United States; ^5^ Department of Nutrition, Exercise and Sports, University of Copenhagen, Copenhagen, Denmark; ^6^ Department of Infectious Diseases, Oslo University Hospital, Oslo, Norway

**Keywords:** TB vaccine, tuberculosis, T-cell responses, transcriptional profiling, AERAS-402, Phase 1 trial

## Abstract

Despite the widespread use of BCG, tuberculosis (TB) remains a global threat. Existing vaccine candidates in clinical trials are designed to replace or boost BCG which does not provide satisfying long-term protection. AERAS-402 is a replication-deficient Ad35 vaccine encoding a fusion protein of the *M. tuberculosis (Mtb)* antigens 85A, 85B, and TB10.4. The present phase I trial assessed the safety and immunogenicity of AERAS-402 in participants living in India – a highly TB-endemic area. Healthy male participants aged 18–45 years with a negative QuantiFERON-TB Gold in-tube test (QFT) were recruited. Enrolled participants (n=12) were randomized 2:1 to receive two intramuscular injections of either AERAS-402 (3 x 10^10^ viral particles [vp]); (n=8) or placebo (n=4) on study days 0 and 28. Safety and immunogenicity parameters were evaluated for up to 182 days post the second injection. Immunogenicity was assessed by a flow cytometry-based intracellular cytokine staining (ICS) assay and transcriptional profiling. The latter was examined using dual-color-Reverse-Transcriptase-Multiplex-Ligation-dependent-Probe-Amplification (dc-RT MLPA) assay. AERAS-402 was well tolerated, and no vaccine-related serious adverse events were recorded. The vaccine-induced CD8^+^ T-cell responses were dominated by cells co-expressing IFN-γ, TNF-α, and IL-2 (“polyfunctional” cells) and were more robust than CD4^+^ T-cell responses. Five genes (*CXCL10, GNLY, IFI35, IL1B* and *PTPRCv2*) were differentially expressed between the AERAS-402-group and the placebo group, suggesting vaccine-induced responses. Further, compared to pre-vaccination, three genes (*CLEC7A, PTPRCv1* and *TAGAP)* were consistently up-regulated following two doses of vaccination in the AERAS-402-group. No safety concerns were observed for AERAS-402 in healthy Indian adult males. The vaccine-induced predominantly polyfunctional CD8^+^ T cells in response to Ag85B, humoral immunity, and altered gene expression profiles in peripheral blood mononuclear cells (PBMCs) indicative of activation of various immunologically relevant biological pathways.

## Introduction

Tuberculosis (TB) remains a major global health challenge. The only TB vaccine in common use, bacillus Calmette-Guérin (BCG), is estimated to reduce the risk of severe TB in children by about 70% (1), but does not prevent contagious TB sufficiently for epidemiological control. A more efficient vaccine would have a large positive impact on global health. Correlates of protection are important to accelerate clinical vaccine development, as they allow much smaller trials of shorter duration to select promising candidates. Currently, no approved correlates of protection exist, but these could be an immunological biomarker or a combination of biomarkers (signature) that are measured in validated assays. Although absolute determinants of protection against *M. tuberculosis* (*Mtb*) are not yet fully understood, T cell immunity is strongly believed to be crucial (2). Therefore, vaccine candidates that induce or boost T cell immunity may hold the key to success. Human *Mtb*-specific CD8^+^ T cells are distinguished by both their preferential recognition of heavily infected cells and restriction by human leukocyte antigen-B (3, 4). Increasing evidence suggests that polyfunctional CD8^+^ T cells, that possess capacities of cytokine production and cytotoxicity (5), have an essential role in the complex interplay resulting in *Mtb* containment and protective immunity. Most vaccine trials have assessed immunogenicity applying T cell stimulation assays with intracellular cytokine staining to characterize changes in vaccine induced CD4 and CD8 T cell populations. However, these assays likely provide limited information on other potentially important immune effects on peripheral blood mononuclear cells [PBMCs] (6).

AERAS-402 is a replication-deficient, adenovirus serotype 35 (Ad35) containing DNA that encodes a fusion protein of three major *Mtb* antigens (Ags) containing both CD4 and CD8 T cell epitopes: Ag85A, Ag85B, and TB10.4 (7–9). Antigen 85A is a 32-kDa protein member of the mycolyl transferase complex involved in cell wall synthesis. It contains several CD4^+^ T cell epitopes and at least one CD8^+^ T cell epitope. Used in a vaccine, Ag85A has protected against *Mtb* challenge in both mice and guinea pigs (10, 11) and is immunogenic in humans (12). Antigen 85B, also referred to as α-antigen, is a 30-kDa mycolyl transferase protein (13, 14), secreted early during *Mtb* infection. It has been previously demonstrated to induce substantial protective immunity against aerosol challenge in the guinea pig TB test system (15). Ag85B is also a component of H4 and H56 subunit vaccines and proved immunogenic in clinical trials of these vaccines (16, 17). Antigen TB10.4 is one of the three members of the very similar ESAT-6 group of proteins found in *Mtb* culture supernatants and known to induce more robust polyfunctional T-cell responses in TB patients compared to *Mtb*-uninfected subjects with/without previous BCG-vaccination (18, 19). A fusion protein of TB10.4 and Ag85B induced a significant additive protective efficacy against aerosol challenge of tuberculosis 10 weeks after immunization of mice (20).

AERAS-402 vaccine phase I trials have previously been conducted in BCG-vaccinated healthy adults in the US (C-001-402, C-008-402 and C-009-402) (21) and South Africa (C-003-402) (22). In these studies, AERAS-402 had a safety profile comparable to other vaccine candidates (21, 23, 24). A robust induction of CD8^+^ T cell responses was observed that appeared to be dose-dependent in some subjects, while CD4^+^ T cell responses were measurable but less prominent (22). Ag85A/B were more immunogenic than TB10.4 (21). Since this study was conducted in 2011, results have been presented in Phase 2 trials evaluating an MVA85A vaccine, a modified Ankara vaccine expressing Ag85A, in infants (C-020-485) (25). H4:IC31, a candidate subunit vaccine that consists of a recombinant fusion protein (H4) and IC31 adjuvant, signaling through toll-like receptor 9 (TLR9), containing mycobacterial antigens Ag85B and TB10.4 in adolescents (C-040-004) (26) neither study showed protection of these antigens against TB infection. Additionally, results from a phase 2 adaptive dose finding study of Aeras 402 (C-029-402) showed that while AERAS-402 had a good safety profile, the immune response was lower than that seen in adults (25).

The objective of the present study (C-004-402) was to evaluate the safety and immunogenicity of the AERAS-402 vaccine for the first time in an Indian population. We performed a phase 1, double-blind, placebo-controlled study. Immunogenicity was assessed by flow cytometry, serology, and a novel high-throughput inexpensive technique for targeted gene expression profiling: Dual-color-Reverse-Transcriptase-Multiplex-Ligation-dependent-Probe-Amplification (dc-RT MLPA).

## Materials and Methods

### Study Approvals and Information on Consent

The study was reviewed and approved by the Government of India Directorate General of Health Services, Office of the Drugs Controller General (Biological Division), Ref no: LL/RA/825/2007 and the Independent Ethics Committee Consultants Bangalore. The phase 1 clinical trial (C-004-402) was registered at (https://clinicaltrials.gov/), and the identifier no: NCT01378312. Written informed consent was obtained from each participant prior to the conduct of any protocol-specific activity or study entry. The study was carried out following the ethical principles outlined in the Declaration of Helsinki and in accordance with the US Code of Federal Regulations for protection of human subjects (21 CFR 50), Institutional Review Boards (21 CFR 56), and the obligations of clinical investigators (21 CFR 312).

### Study Design, Enrolment, and Vaccination

**Figure 1:**
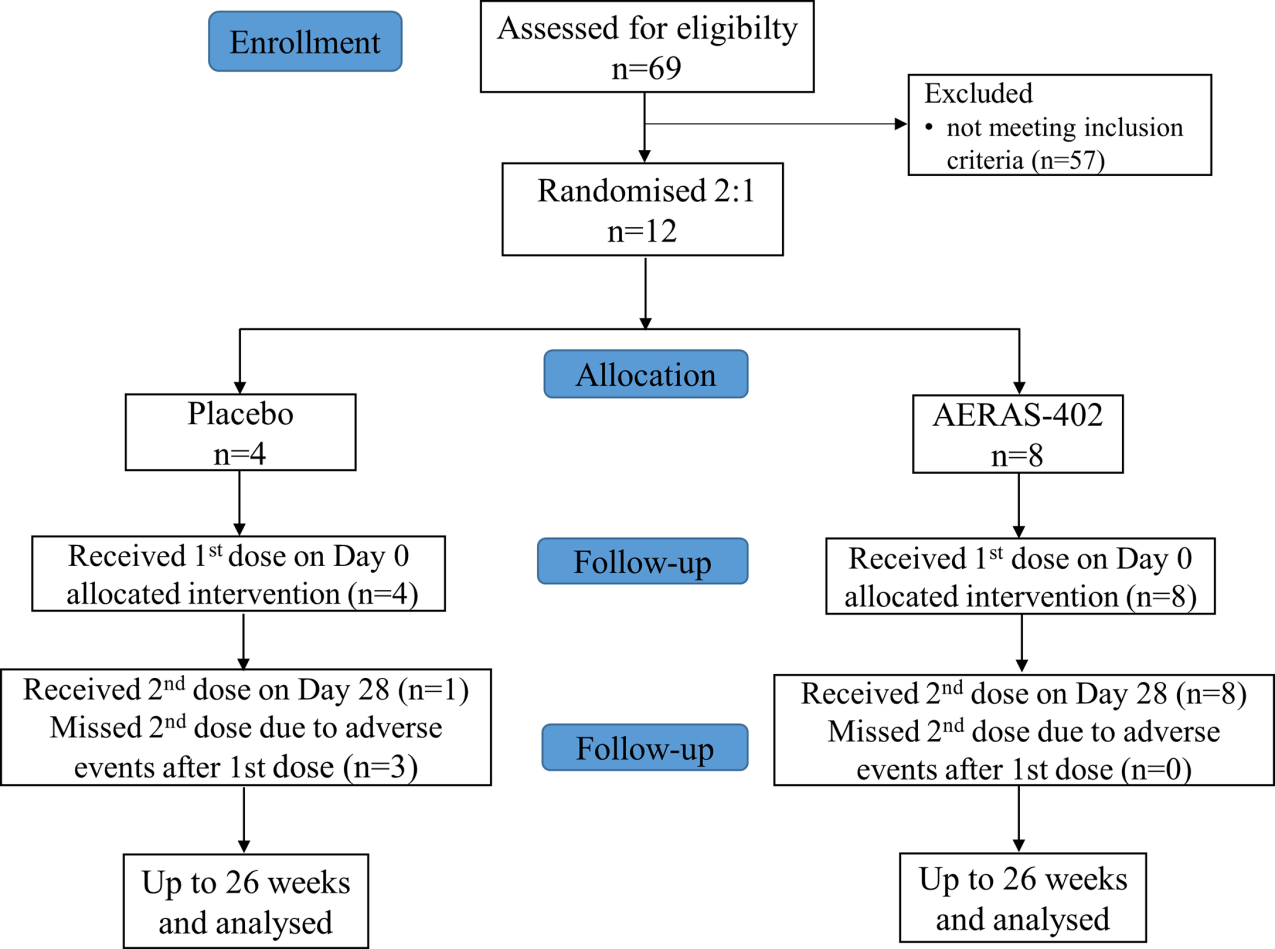
Study flow chart.

The study was conducted at Lotus lab, Bangalore, India (12.9303° N, 77.6217° E) between February 2011 and October 2011. The major inclusion criteria were i) age 18 through 45 years on Study Day 0, ii) had BCG vaccination at least 5 years ago, iii) documented through medical history or presence of scar, iv) has Body Mass Index (BMI) between 19 and 30 (kg/m^2^), v) has ability to complete follow-up period of 182 days as required by the protocol, and vi) has completed written informed consent. The major exclusion criteria were i) acute illness on the day of randomization, ii) oral temperature ≥37.5°C on the day of randomization, iii) used immunosuppressive medication/received immunoglobulin or blood products/received any standard vaccine within 42 days, iv) history or evidence (including chest X-ray) of active tuberculosis, tuberculin skin test evidence of *Mtb* infection, defined as 15 mm of induration or greater and laboratory test evidence of *Mtb* infection, v) abnormal laboratory parameters and use of intravenous drugs. Enrolled subjects were randomized 2:1 to receive two intramuscular injections of either AERAS-402 (3 x 10^10^ viral particles [vp]) or placebo (normal saline solution) on study days 0 and 28 (**Figure 1**).

The sample size for this study was selected as adequate for an initial review of the developing safety profile of AERAS-402 in a BCG-vaccinated population at the selected dose level, rather than for statistical reasons. A sample size of eight subjects in the AERAS-402 treatment group permitted initial estimates of the incidence of adverse events; given eight subjects receiving AERAS-402, this study had an 80% or greater chance of detecting an adverse event with a rate of occurrence of 18% in the study population under consideration.

### Follow Up and Safety Evaluations

Subjects had vital signs (blood pressure, pulse, and oral temperature) measured just before receiving each vaccination with AERAS-402 or placebo and at 30 minutes and 60 minutes post vaccination, and two days after vaccination. Blood was collected for routine clinical chemistry and hematology at screening and post vaccination. Vaccination was administered on days 0 and 28, and safety and immunogenicity were assessed on days 0, 7, 14, 28, 35, 42, 56 and 182.

Adverse events (AEs) were collected for 28 days after each immunization and solicited AEs, including assessment for local injection site reactions (pain, redness and swelling at the site of injection; arthralgia; conjunctivitis; diarrhea; dysuria; fatigue; fever; headache; malaise; myalgia; sore throat; and upper respiratory tract infection) were recorded by subjects on diary cards for 14 days after each vaccination. Serious adverse events were collected from the time of first study vaccine dosing through study day 182. Adverse events were graded by severity and relationship to study vaccine using predefined criteria.

### Tuberculin Skin Test (TST) and QuantiFERON-TB Gold

TST (measured in millimeters at the transverse induration) and QFT- TB Gold tests were conducted at screening and at study day 182.

### Peripheral Blood Mononuclear Cell (PBMC) Intracellular Cytokine Staining (ICS) Assay and Flow Cytometry

PBMCs were sent from Bangalore to Aeras Rockville, MD, USA. The PBMC ICS assay was performed as previously described (27). Briefly, PBMCs were thawed and rested overnight and then stimulated for 5-6 hours at 37°C with 0.4% Dimethyl Sulfoxide (DMSO; negative control), 0.5ug/mL Staphylococcal enterotoxin B (SEB; positive control) or with peptide pools, one pool per antigen (1 µg/peptide/mL with pools for Ag85A, Ag85B and TB10.4), in the presence of Brefedin A and monensin (GolgiStop and GolgiPlug), both from BD Biosciences USA and used at 1 µL per well. Peptide pools were 15mers overlapping by 11 amino acids and spanning the sequences of the vaccine encoded antigens, Cells were then stained using viability dye (Live/Dead Aqua, Invitrogen, USA), anti-CD4-V450, and anti-CD8 PE-Cy5, then fixed and permeabilized for intracellular staining using anti-CD3 APC-Cy7, anti-IFN-γ APC, anti-TNF-α FITC, and anti-IL-2 PE (all antibodies from BD Biosciences, USA). Data were acquired using a BD LSRII flow cytometer (BD Biosciences, USA) and analyzed using FlowJo software (TreeStar Inc., USA). Immunogenicity was determined by first gating on live, singlet CD3+ T cells and then gating CD4+ and CD8+ T cells and calculating the percentage of each population that was cytokine positive. DMSO subtraction was performed prior to plotting the results. The gating strategy for vaccinated and placebo recipients are provided as a **Supplementary Figure 1**.

### Anti-Mycobacterial Antibodies by ELISA

The serum samples were tested at 1:100 dilution to measure antigen-specific antibodies by ELISA. Briefly, the ELISA was performed as follows: recombinant *Mtb* antigen Ag85B purified in-house at Aeras was coated at 1.5 µg/ml onto 96-well ELISA plates. The immobilized antigen was incubated with 100 µl of serum samples at 1:100 dilution to capture antigen-specific antibodies. The captured antibodies were then probed by the addition of 100 µl of biotinylated anti-IgG antibodies at 1:500 dilution and detected by adding 100 µl of colorimetric substrate solution. The substrate color development was stopped using 50 µl of stop solution and the color intensity was read using a spectrophotometer and analyzed with SoftMax^®^ Pro 5.4.1 data acquisition & analysis software.

### Adenovirus 35 Neutralization

Adenovirus 35 serum neutralization activity was assayed pre-vaccination with placebo or AERAS-402 at study day 0, and at study day 182. Briefly, neutralizing antibody titers against Adenovirus type 35 were determined using the validated neutralizing antibody assay at Crucell using a previously published method (28). Luminescence counts were recorded on a 1450 Micro Beta Trilux. Data were imported into MS Excel to calculate 90% inhibition titers.

### Sample Preparation and RNA Extraction

An aliquot of frozen PBMCs were shipped from Aeras Rockville, MD, USA to Bergen, Norway for the dcRT-MLPA assay. The PBMCs were thawed at 37° C water bath and ~2 million cells (avg: 4.45, min: 0.2, max: 15.73, SD: 3.19) were immediately transferred into 1.7ml sterile RNase-free tubes containing 1ml of RNAlater^®^: RNA stabilization solution (ThermoFisher Scientific). Following incubation at room temperature for 1 hours, subsequently the samples were stored at -70°C for further analysis.

Total RNA was extracted from the PBMCs using the RNeasy Mini Kit (Qiagen, Hilden, Germany) with RNase free DNase on-column digestion (Qiagen, Hilden, Germany) according to the manufacturer’s instructions. The total RNA concentration and purity (A260/280 nm ratio) were measured using a Nanodrop spectrophotometer (Thermoscientific, Wilmington, Delaware, U.S.A).

### Dual-color Reverse Transcriptase-Multiplex Ligation-Dependent Probe Amplification (dcRT-MLPA)

We used a novel high-throughput technique, which requires only ~125 ng of total RNA for analyzing a predefined panel of genes of interest. RNA samples from 12 subjects: 4 received placebo, and 8 received AERAS-402 vaccine (PBMC samples from six time points, i.e., days 0, 7, 28, 35, 42, and 182) were used for dcRT-MLPA analysis. A modified one-step protocol of dcRT-MLPA was used as previously described (29). A total of 150 genes (including 4 housekeeping genes), distributed in two panels were assessed, based primarily on their posited or confirmed roles in TB immunology. The first gene set contains 58 genes that included type-1 interferon-inducible genes (30) known to be upregulated in adult TB and genes associated with predicted risk for TB in South African neonates (31); the innate and adaptive gene set contains 92 genes which known for involvement in general inflammation and myeloid cell activation, and genes involved in the adaptive immune system, comprising Th1/Th2-responses, regulatory T-cell markers and B-cell associated genes (32). DcRT-MLPA probes and primers (reverse transcription gene target-specific primers, right- and left-hand half MLPA probes, FAM labelled MLPA primers, HEX labelled MAPH primers) were obtained from the Department of Infectious Diseases, Leiden Medical University, Leiden, The Netherlands. The dcRT-MLPA reagents were purchased from MRC Holland, The Netherlands. Samples with a concentration <50 ng µl^-1^ were concentrated at 45°C using a speed vacuum concentrator (Eppendorf AG, Hamburg, Germany). A positive control for each gene panel (using synthetic template oligonucleotides as hybridization templates) and a commercial Human Universal Reference RNA were included on each plate. The amplified PCR products were diluted 1:10 with nuclease-free water and added to a mixture of Hi-Di-Formamide with 400HD ROX size standard. The PCR products were denatured at 95°C for 5 minutes and then immediately cooled on ice. Subsequently, the PCR fragments were analyzed on a 3730-capillary sequencer in Gene scan mode (Life Technologies, Carlsbad, California, USA).

### DcRT-MLPA Data Processing

Data were analyzed using GeneMapper software version 5.0 (Life Technologies, Carlsbad, California, USA). The default peak detection settings were inspected and adjusted if necessary. The peak area (in arbitrary units) was normalized against GAPDH using Microsoft Excel spreadsheet software. The genes that had no or little expression (peak area < 200 arbitrary units) were assigned to a threshold value of 200 arbitrary units.

### Statistical Methods

Descriptive statistics were performed to summarize AERAS-402 adverse events and immunogenicity. Mann-Whitney test (placebo vs. vaccinated) and paired t-test (pre vaccination vs. post vaccination) analysis were performed where appropriate. No multiplicity adjustment of p-values was applied. Mean difference and corresponding standard errors were reported. IBM SPSS software version 24.0 (IBM, Bergen, Norway) was used. Dot plots were created using GraphPad Prism 8 (GraphPad software, San Diego, CA). WebGestalt (WEB-based GEne SeT AnaLysis Toolkit) (33) is one of the most widely used gene set enrichment analysis tools that help users extract biological insights from genes of interest. WebGEStalt and the Functional Enrichment Analysis (FunRich) tool (34) was applied for the gene enrichment and network pathway analysis. The top results were ranked using the Benjamini–Hochberg method for controlling the false discovery rate. A p-value < 0.05 was considered significant.

## Results

### Subject Demographics and Vaccination

Between Feb 2011 and April 2011, 69 subjects were screened and 12 recruited and randomized to the AERAS-402 group (n=8) or placebo (n=4). Demographic and other baseline characteristics were well balanced between the groups (**Table 1**). All 8 subjects in the AERAS-402 group and one subject in the placebo group received both study vaccinations; the remaining 3 subjects in the placebo group did not receive the second vaccination due to adverse events. All 12 subjects completed the study follow up period of 182 days.

**Table 1:** Demographic and Baseline Characteristics.

Parameter	Placebo 2 Doses	AERAS-402 (3 x 10^10^ vp) 2 Doses	Total
	(n=4)	(n=8)	(n=12)
**Age (years)**			
n	4	8	12
Mean	28.3	27.4	27.7
**Age Group (years), n (%)**			
18-30	4 (100.0)	7 (87.5)	11 (91.7)
31-40	0 (0.0)	1 (12.5)	1 (8.3)
**Gender, n (%)**			
Male	4 (100.0)	8 (100.0)	12 (100.0)
**Race, n (%)**			
Indian	4 (100.0)	8 (100.0)	12 (100.0)
**Height (cm)**			
n	4	8	12
Mean	164.80	168.55	167.30
**Weight (kg)**			
n	4	8	12
Mean	62.28	69.50	67.09
**Body Mass Index (kg/m^2^)**			
n	4	8	12
Mean	22.97	24.47	23.97
**Documentation of BCG Vaccination, n (%)**			
Medical history	4 (100.0)	8 (100.0)	12 (100.0)
Presence of scar	4 (100.0)	8 (100.0)	12 (100.0)

### AERAS-402 Safety

No serious adverse events were reported. All 8 subjects in the AERAS-402-group and 4 subjects in the placebo-group reported at least 1 AE after either the study day 0 or study day 28 vaccination, and the majority of AEs were mild-moderate (**Table 2**). One subject in the AERAS-402-group had severe AEs (transient injection site pain, myalgia, and fatigue after the study day 0 vaccination) considered related to the study vaccine, but all AEs resolved within 7 days. Two AEs were reported for more than 1 subject in both intervention groups: decreased hemoglobin (reported for all subjects in both treatment groups) and injection site pain (5 in the AERAS-402 group vs. 0 in the placebo group). Note that in this study, decrease of any magnitude in hemoglobin from baseline was recorded as an AE.

**Table 2:** Number of subjects (%) with AEs for study day 0 or 28 post-vaccination.

Solicited/Unsolicited events	Placebo 2 Doses	AERAS-402 (3 x 10^10^ vp) 2 Doses
	(n=4) (%)	(n=8) (%)
Subjects with at least 1 adverse event	4 (100.0)	8 (100.0)
Investigations	4 (100.0)	8 (100.0)
Blood creatine phosphokinase increased	1 (25.0)	0 (0.0)
Blood creatinine increased	1 (25.0)	0 (0.0)
Hemoglobin decreased	4 (100.0)	8 (100.0)
Lymphocytes count increased	1 (25.0)	0 (0.0)
Neutrophils count decreased	0 (0.0)	1 (12.5)
Neutrophils count increased	0 (0.0)	1 (12.5)
White blood cells count increased	0 (0.0)	1 (12.5)
General disorders and administration site conditions	0 (0.0)	5 (62.5)
Gastrointestinal disorders	0 (0.0)	1 (12.5)
Musculoskeletal and connective tissue disorders	0 (0.0)	1 (12.5)
Respiratory, thoracic and mediastinal disorders	0 (0.0)	1 (12.5)
Renal and urinary disorders	1 (25.0)	0 (0.0)

The 3 subjects, in the placebo group that did not receive their second vaccination due to the following abnormal laboratory values at Study Day 28:

- Grade 1 hematuria which was considered unlikely to be related to study vaccine.
- Grade 3 CPK which was considered unlikely to be related to study vaccine.
- Grade 1 decreased hemoglobin which was considered not related to study vaccine.

These abnormal laboratory values were reported as adverse events. Due to the exclusion criteria specifying that laboratory values were required to be within local laboratory normal ranges in order to receive the Study Day 28 vaccination, all three subjects did not receive the second dose of study vaccine, but were followed to study completion, Study Day 182.

### AERAS-402 Administered at Two Doses Induces Polyfunctional CD8^+^ T Cell Responses

**Figure 2:**
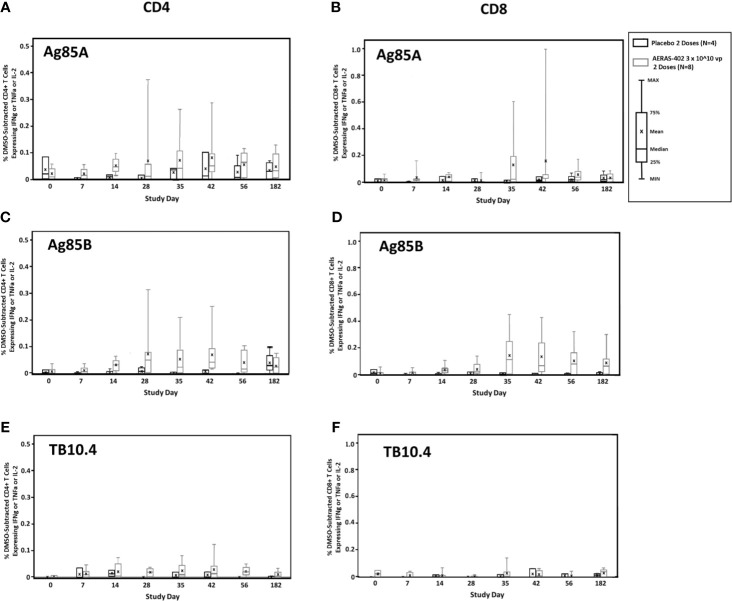
T cell responses to vaccine-encoded antigens. PBMCs were thawed, rested overnight, and stimulated for 5-6 hours with DMSO (negative control), SEB (positive control), or peptide pools corresponding to the vaccine antigens Ag85A **(A, B)**, Ag85B **(C, D)**, or TB10.4 **(E, F)**. Specimens were then stained for viability, phenotypic markers, and intracellular cytokine expression and evaluated by flow cytometry. Data were analyzed using FlowJo software to generate cytokine Boolean gates. Each gate was subjected to DMSO subtraction to remove background. Negative results following DMSO-subtraction were set to zero. The sum of these gates was then used to determine the total cytokine response for CD4+ **(A, C, E)** and CD8+ **(B, D, F)** T cells for the AERAS-402-vaccinated (grey bars) or placebo control (black bars) groups. Bars are plotted for each group and time point for the total response (any cytokine alone or in combination for IFN-*γ* or IL-2 or TNF-α). Bars represent the 25^th^ to 75^th^ percentile, with the cross bar representing the median response. The mean is indicated by an “x” and the error bars represent the minimum and maximum responses.

Antigen-specific CD4^+^ and CD8^+^ expression of the cytokines IFN-*γ*, TNF-α, and/or IL-2 alone or in combination to the individual AERAS-402 antigens, Ag85A, Ag85B, and TB10.4, are presented by the intervention group in **Figures 2A–F**. AERAS-402 induced responses to Ag85B constituted the most convincing differences between the AERAS-402-group and the placebo-group. AERAS-402 induced vaccine specific CD4^+^ and CD8^+^ T-cell response (mean response) peaked at day-35 and -42 of post vaccination of both doses. However, the magnitude of CD8^+^ T cell responses was in general higher than CD4 T cell responses. Vaccine-induced responses peaked at study days 35 and 42 (7 and 14 days after the second dose of vaccination) and were in some cases, sustained through study day 182.

**Figure 3:**
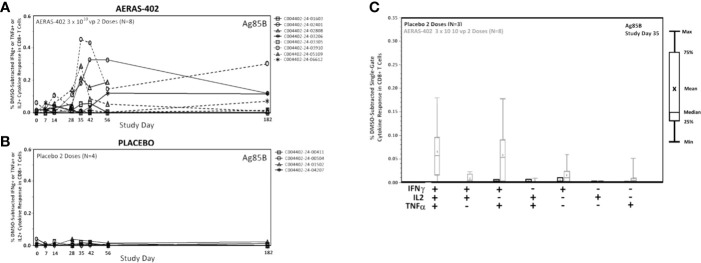
CD8^+^ cytokine responses to Ag85B. PBMCs were thawed, rested overnight, and stimulated for 5-6 hours with DMSO (negative control), SEB (positive control), or peptide pools corresponding to the vaccine antigens Ag85A, Ag85B, or TB10.4. Specimens were then stained for viability, phenotypic markers, and intracellular cytokine expression and evaluated by flow cytometry. Data was analyzed using Flow Jo software to generate cytokine Boolean gates. Each gate was subjected to DMSO subtraction to remove background. Negative results following background subtraction are set to zero. The sum of these gates was then used to determine the total cytokine response for vaccinated **(A)** and placebo control **(B)** groups or plotted by functionality to assess the polyfunctional response **(C)**. Lines **(A, B)** are plotted for each individual subject. CD8^+^ Single-Gate (Boolean; **C**) ICS responses for Ag85B at Study Day are shown for vaccinated (gray bars) and placebo (black bars) groups. Bars represent the 25^th^ to 75^th^ percentile, with the cross bar representing the median response. The Mean is indicated by an “x” and the error bars represent the minimum and maximum responses.

Frequencies of Ag85B-specific CD8 T cell >0.05% at day 35 and 42, were observed in 7/8 (87.5%) in the AERAS-402-group compared to 0/4 (0%) in the placebo-group (**Supplementary Figure 2**). Individual trajectories stratified for intervention group are demonstrated in **Figures 3A, B**. The most prominent CD8^+^ T cell subsets (Boolean gating) to Ag85B at study day 35 were the polyfunctional IFN-*γ* ^+^IL-2^+^TNF-α^+^ and bifunctional IFN-*γ* ^+^TNF-α^+^ subsets (**Figure 3C**).

### AERAS-402 Administered at Two Doses Boosts Specific Antibody Production

Ag85B antibodies were detected in the AERAS-402-group with peak responses at study days 42 and 56. Ag85B antibodies were not measurable in the placebo-group (**Supplementary Figure 3**).

### Anti-Adenovirus 35 Antibodies

Pre-existing antibodies towards adenovirus could affect vaccine efficacy. Therefore, Adenovirus 35 serum neutralizing activity was assessed pre-vaccination. A response >LLOQ for Adenovirus 35 serum neutralizing activity was detected in 2/8 in the AERAS-402-group and 3/4 in the placebo group (**Table 3**). At study day 182 of the six subjects in the AERAS-402 recipients with neutralizing activity ≤ LLOQ at study day 0, two subjects had a shift in Adenovirus 35 neutralizing activity response to >LLOQ.

**Table 3:** AERAS-402 anti-Ad35 Neutralizing Activity.

Parameter/Study Day (Time)	Placebo 2 Doses	AERAS-402 (3 x 10^10 vp) 2 Doses
	(n=4)	(n=8)
**Response > LLOQ, n (%)**		
Study Day 0 (Pre-Vaccination)	3 (75.0)	2 (25.0)
Study Day 182	3 (75.0)	4 (50.0)
**Shift from Study Day 0 (Pre-Vaccination) to Study Day 182 (%)**		
> LLOQ to > LLOQ	3/4 (75.0)	2/8 (25.0)
> LLOQ to <= LLOQ	0/4 (0.0)	0/8 (0.0)
<= LLOQ to <= LLOQ	1/4 (25.0)	4/8 (50.0)
<= LLOQ to > LLOQ	0/4 (0.0)	2/8 (25.0)

### Similar TST and QFT-G Responses Following Vaccination

For all 12 subjects, the TST induration and QFT were measured on screening day and study day 182. The TST mean induration decreased from screening to study day 182 in both groups (**Table 4**). None of the study participants had QFT conversion at day 182.

**Table 4:** Tuberculin Skin Test (TST) results.

Study Day (Time)/Parameter	Placebo2 Doses	AERAS-402(3 x 10^10^ vp)2 Doses
	(n=4)	(n=8)
**Screening**		
n	4	8
Mean	7.3 ± 4.5	6.9 ± 3.0
0-10 mm, n (%)	3 (75.0)	7 (87.5)
11-15 mm, n (%)	1 (25.0)	1 (12.5)
**Study Day 182**		
n	4	8
Mean	5.3 ± 3.86	3.9 ± 0.83
0-10 mm, n (%)	3 (75.0)	8 (100.0)
11-15 mm, n (%)	1 (25.0)	0 (0.0)
**Shift from screening to study day 182, (%)**		
0-10 mm to 0-10 mm	3/4 (75.0)	7/8 (87.5)
11-15 mm to 0-10 mm	0/4 (0.0)	1/8 (12.5)
11-15 mm to 11-15 mm	1/4 (25.0)	0/8 (0.0)

### AERAS-402 Induced Gene Expression in PBMCs

**Figure 4:**
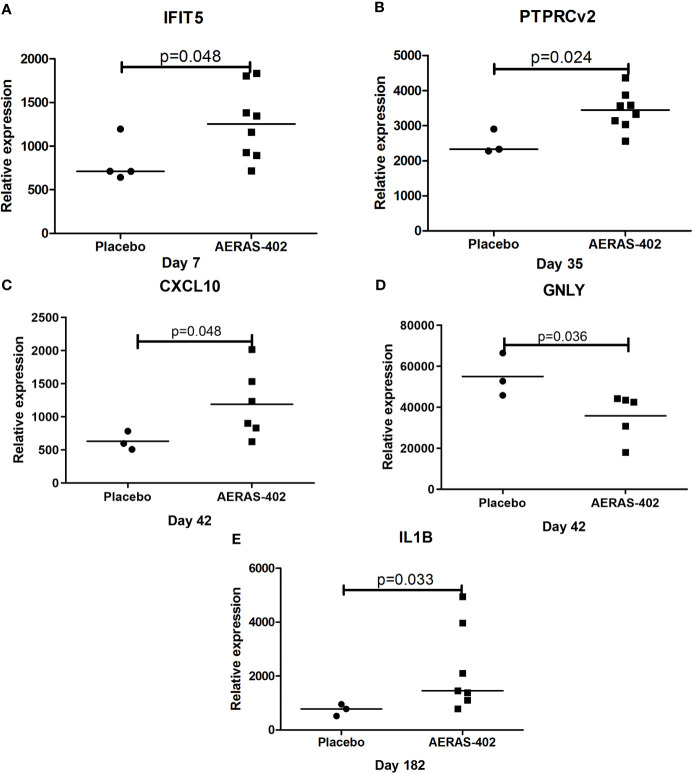
Mann-Whitney test was applied. Dot-plot graph depicting genes that were differentially expressed between the AERAS-402 and placebo recipients. Day 7 **(A)**; day 35 **(B)**; day 42 **(C, D)**; day 182 **(E)**.

Gene expression levels for 7/46 genes in the type-1 interferon inducible gene set and 24/85 genes in the innate and adaptive gene set, were undetectable in all 63 samples. For the remaining genes with detectable levels, results of the comparison of gene expression between the AERAS-402-group and the placebo-group are shown in **Tables 5A**, **B**. Post-vaccination at day 7, day 35 and day 182, transcription of *IFIT5, PTPRCv2* and *IL1B* was upregulated (all p<0.05) in the AERAS-402-group compared to the placebo-group (**Figures 4A, B, E**). At day 42, the transcription of *CXCL10* was upregulated (p=0.048) and the transcription of *GNLY* down-regulated (p=0.036) in the AERAS-402-group compared to the placebo-group (**Figures 4C, D**).

**Table 5a:** Comparison of baseline measurements of each biomarkers that changed with post-vaccination of AERAS-402.

	Genes	day 0 and day 7^Ψ^			day 0 and day 28^Ψ^			day 0 and day 35^Ψ^			day 0 and day 42^¥^			day 0 and day 182^¥^		
		Mean difference	SE Mean	p-value	Mean difference	SE Mean	p-value	Mean difference	SE Mean	p-value	Mean difference	SE Mean	p-value	Mean difference	SE Mean	p-value
Type-1 IFN inducible	**CXCL10**	451.7	367.9	0.265	859.4	537.4	0.161	142.7	293.0	0.643	268.4	841.1	0.470	587.5	78.6	**0.001**
	**IFI35**	468.4	873.0	0.611	1671.3	1283.8	0.241	-617.0	1076.7	0.578	305.1	1077.2	0.789	2702.7	895.7	**0.030**
	**IFIT3**	683.6	469.5	0.196	576.0	357.1	0.158	919.4	1218.4	0.479	118.0	476.1	0.815	1424.2	431.0	**0.021**
	**TAP2**	232.1	304.9	0.475	513.7	200.4	**0.043**	250.0	248.1	0.353	-127.0	246.4	0.626	22.7	345.4	0.950
Hanekom set	**BMP6**	1838.4	1822.5	0.352	995.7	1243.5	0.454	345.4	1058.1	0.755	1412.2	1173.3	0.909	3552.0	1134.9	**0.026**
	**GUSB**	293.7	257.6	0.298	376.1	218.8	0.136	131.6	163.3	0.451	137.1	148.2	0.399	818.8	287.3	**0.036**
	**KIF1B**	491.9	260.8	0.108	324.7	225.0	0.199	268.4	140.6	0.105	122.3	191.1	0.552	457.7	154.3	**0.031**
	**LYN**	383.9	162.2	0.056	1336.7	383.2	**0.013**	655.3	291.2	0.065	711.2	336.4	0.088	1796.5	1036.8	0.144
	**VEGF**	-18.4	21.0	0.413	133.1	86.8	0.176	130.4	51.6	**0.045**	276.3	64.3	**0.008**	-29.8	39.5	0.484
Innate and adaptive	**CCL5**	6127.4	2222.7	**0.033**	2371.1	1947.8	0.269	3833.6	1562.6	**0.050**	-544.1	1364.4	0.710	3306.2	2832.9	0.296
	**CCR7**	715.3	349.6	0.087	450.3	216.6	0.083	1173.3	336.8	**0.013**	446.4	393.2	0.320	319.8	326.5	0.372
	**CD3E**	3363.9	1015.1	**0.016**	2700.3	885.1	**0.022**	2821.0	954.2	**0.025**	671.0	1066.1	0.564	1407.0	867.2	0.166
	**CD8A**	979.9	557.8	0.129	580.9	480.6	0.272	1470.4	515.5	**0.029**	37.6	586.2	0.952	497.2	781.8	0.553
	**CLEC7A**	1627.7	696.3	0.056	1932.7	800.4	**0.050**	1372.4	407.9	**0.015**	1213.6	409.3	**0.041**	1907.2	595.9	**0.024**
	**CXCL13**	581.1	185.3	**0.020**	313.6	163.8	0.104	312.0	88.6	**0.012**	290.0	229.4	0.275	373.8	169.6	0.079
	**GATA3**	179.9	65.5	**0.033**	122.1	41.4	**0.026**	83.3	55.6	0.185	68.8	78.2	0.429	45.7	63.0	0.501
	**GZMA**	-734.4	720.0	0.347	-2111.6	542.5	**0.008**	-1690.9	688.0	**0.049**	-2813.0	502.2	**0.005**	821.8	1800.9	0.667
	**NLRC4**	-128.1	154.4	0.438	-81.1	50.8	0.161	-358.4	110.7	**0.018**	389.0	452.4	0.438	417.0	321.4	0.251
	**NLRP1**	2473.6	1179.9	0.081	2599.7	692.8	**0.009**	2436.4	406.2	**0.001**	1425.4	940.8	0.240	1971.8	1466.9	0.237
	**NLRP3**	166.0	148.5	0.306	674.3	197.6	**0.014**	389.9	172.6	**0.001**	752.9	89.8	**0.001**	63.7	177.6	0.735
	**NOD1**	321.9	169.7	0.107	390.9	153.2	**0.043**	256.0	66.3	**0.008**	46.6	264.7	0.869	258.3	143.2	0.131
	**PTPRCv1**	3436.4	862.3	**0.007**	2378.7	833.5	**0.029**	2426.3	622.1	**0.008**	1613.6	914.0	0.152	2243.3	847.3	**0.046**
	**PTPRCv2**	927.0	359.7	**0.042**	709.6	371.1	0.104	791.6	170.3	**0.004**	-145.0	370.2	0.716	769.8	504.9	0.188
	**TAGAP**	2718.0	809.5	**0.015**	1475.1	467.7	**0.020**	2064.0	568.9	**0.011**	356.8	604.1	0.587	1906.0	713.8	**0.044**
	**TLR4**	-26.9	53.8	0.636	77.6	38.2	0.088	-77.3	68.3	0.301	131.2	148.0	0.428	340.0	124.1	**0.041**
	**TLR7**	-763.0	304.2	**0.046**	-223.0	327.8	0.522	-603.1	315.6	0.105	-257.4	682.3	0.725	290.7	193.3	0.193
	**ZNF331**	437.0	244.6	0.124	210.4	100.0	0.080	920.1	300.4	**0.022**	741.0	192.4	**0.018**	17.3	30.6	0.596

Ψ - 7 subjects; ¥ - 6 subjects. SE, Standard error; p-value obtained based on paired t-test. The significant p-values are in bold.

**Table 5b:** Comparison of baseline measurements of each biomarkers that changed with post-vaccination of placebo.

	Genes	day 0 and day 7^Ψ^			day 0 and day 28^Ψ^			day 0 and day 35^Ψ^			day 0 and day 42^¥^			day 0 and day 182^¥^		
		Mean difference	SE Mean	p-value	Mean difference	SE Mean	p-value	Mean difference	SE Mean	p-value	Mean difference	SE Mean	p-value	Mean difference	SE Mean	p-value
Type-1 IFN inducible	**IFI6**	1660.33	562.55	0.098	1118.67	88.83	**0.006**	718.33	387.76	0.205	1650.67	870.80	0.198	1888.00	285.85	**0.022**
	**IFIT5**	53.33	242.18	0.846	381.33	64.06	**0.027**	107.00	69.35	0.263	104.67	115.51	0.461	483.67	235.96	0.177
	**IFI44**	464.33	601.68	0.521	896.66	255.9	0.073	187.67	44.82	**0.050**	113.06	115.47	0.432	770.00	341.56	0.153
	**IFI44L**	175.00	264.92	0.577	370.67	85.49	**0.049**	-22.00	156.04	0.901	189.33	109.41	0.226	250.33	255.65	0.431
	**OAS3**	-109.67	75.65	0.284	21.67	138.15	0.89	-211.33	43.23	**0.039**	59.33	152.09	0.734	-101.33	163.68	0.599
	** **															
Innate immunity	**NOD2**	23.00	66.00	0.787	96.5	7.5	**0.049**	49.50	0.50	**0.006**	31.33	75.01	0.750	14.00	57.00	0.847

₳3 subjects; SE, Standard error; p-value obtained based on paired t-test. The significant p-values are in bold.

**Figure 5:**
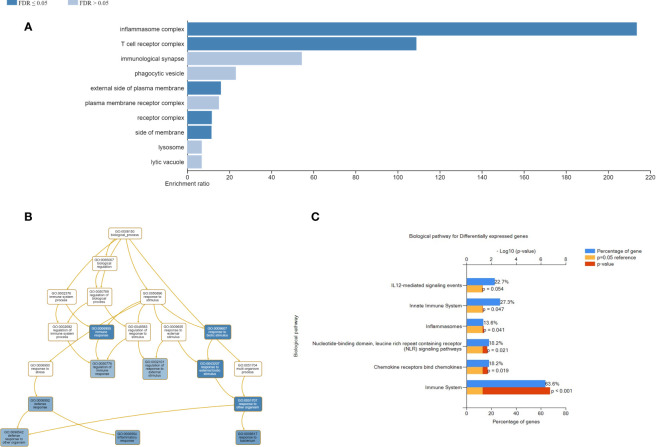
Improved visualizations on the result page of WebGestalt. **(A)** Bar chart shows enrichment ratio or normalized enrichment score of results with direction. **(B)** Directed acyclic graph representation with colored nodes depicting corresponding biological process of the enriched genes in the input gene set. Functional enrichment analysis of genes using FunRich. **(C)** Bar graph of biological pathways in percentage of genes are shown; the blue bar represents the percentage of genes, the yellow bar represents the reference p-value=0.05 and the red bar that depicts the exact p-value.

We then assessed the change in gene expression profiles from baseline up to day 182 within each intervention group (**Tables 5A**, **B**). Among 132 unique genes, 33 genes were differentially expressed in the vaccination and placebo groups and were selected for functional categories and used for the enrichment analysis. WebGestalt identified the most significant gene sets and showed that the inflammasome and T cell receptor complexes were significantly ranked higher than other complexes. The Gene Ontology enrichment in the biological process is highlighted with colors based on FDR significance (**Figure 5A**). Each ontology consists of a set of gene ontology (GO) terms, which are organised in a hierarchy, or directed acyclic graph (DAG), as shown in **Figure 5B**. As expected by the fact that AERAS-402 was designed to induce T cell responses, signaling related to the T cell receptor are engaged. In addition, the FunRich arranged the differentially expressed genes into two functional categories, including biological processes and cellular components. The parameters for the interception of biological pathways are indicated in **Figure 5C**.

### Comparison of Gene Expression Profiles Between Pre and Post Vaccination

The gene expression profiling shows that compared to pre-vaccination, the innate immune genes (*CLEC7A* and *NLRP3)*; T-cell associated genes (*PTPRCv1* and *TAGAP*) were consistently upregulated (all p ≤ 0.05) at 4- time points in the AERAS-402-group (**Table 5A**). Next, compared to pre-vaccination, on day-7 after the first and second dose of vaccination (i.e., day-7 and day-35, respectively), the innate immune genes (*CCL5, CXCL13*) and T-cell subset specific genes (*CD3E, PTPRCv2* [CD45RO]) were upregulated (all p<0.05). Similarly, on day-7 after the second dose of vaccination (i.e., day-35; **Table 5A**), the innate immune response NLR and NOD-like specific genes (*NLRP1, NLRP3*, and *NOD1*) were upregulated (all p<0.01). Correspondingly, at day-35 and -42, following the second vaccination, three genes that are involved in innate immunity (*NLRP3*, *VEGF* and *ZNF331;* p<0.05) were up-regulated, whereas the T-cell associated *GZMA* was downregulated (p<0.05). The changes observed in gene expression profiles suggest broad vaccine-induced changes. Notably, none of these changes were observed in the placebo-group (**Table 5B**).

## Discussion

In line with previous phase 1 trials of AERAS-402 in other populations (22), we report an acceptable safety profile for this vaccine candidate in healthy BCG vaccinated *Mtb–*uninfected adults. The vaccine also induced promising immunogenicity, with robust polyfunctional T-cell responses to Ag85B predominantly in CD8 subsets that peaks at day 35 and 42, corresponding to one week following the second vaccination. There were no serious adverse events that were related to the vaccine, mild grade abnormal hematology was seen in AERAS-402 candidates.

CD4^+^ T cells play a central role in TB protective immunity (35, 36). Consistent with the previous report (22), AERAS-402 induced a vaccine-specific CD4^+^ T-cell response, which was dominated by the polyfunctional IFN-γ^+^TNF-α^+^IL-2^+^ subset to all antigens (**Figures 2**, **3**). The results seen in this study are consistent with previous studies demonstrating that the vaccine-induced a robust CD8^+^ T-cell response against Ag85A, Ag85B and TB10.4 (**Figure 2**) (22). Nevertheless, to date, most new TB vaccines have been reported to induce reasonable CD4^+^ T-cell responses, but relatively negligible CD8^+^ T-cell responses, despite evidence from recent studies indicating that CD8^+^ T cells mediate essential roles in protective immunity against TB (5, 37–39) including cytolytic functions to kill *Mtb*-infected cells *via* granule-mediated function (via perforin, granzymes, and granulysin) and Fas-Fas ligand interaction to induce apoptosis. In humans, CD8^+^ T cells can produce granulysin, which can kill *Mtb* directly (40). Despite having the same or similar antigens, the immune response that is generated against the TB antigens can vary greatly based on the method of delivery. For example, protein/adjuvant combinations drive primarily CD4+ T cell responses and antibody responses. However, vaccines that include adenoviral vectors are better suited for stronger induction of the cellular arm of the adaptive immune system, including CD8+ cytotoxic T cells.

We report that the present study is the first TB clinical vaccine phase I trial to assess vaccine induced PBMC transcriptomes. Compared to the placebo group, two genes (*IFIT5* and *PTPRCv2*) were upregulated in the 7-days following the 1^st^ and 2^nd^ dose of vaccination, respectively, while *CXCL10* and *GNLY* were differentially expressed between the intervention groups 14-days following the 2^nd^ dose of vaccination. Interestingly, 6 months post-vaccination *IL1B* was upregulated in the AERAS 402 group. These genes are evocative of robust CD4^+^ and CD8^+^ T cell responses as observed in the T-cell stimulation assay, which likely includes activation of the type-1 interferon and cytotoxicity genes. Besides, we have identified three genes (*CLEC7A, PTPRCv1*, and *TAGAP)* that are consistently induced following two doses of AERAS-402: The C-type lectin receptors are a class of signaling pattern recognition receptors, macrophages, neutrophils and dendritic cells express CLEC7A. The innate immunity component CLEC7A (Dectin-1) interacts with *Mtb*, leading to increased inflammatory cytokine production in macrophages (41, 42). The T-cell associated gene *PTPRCv1*(CD45RA) is expressed on naive T cells, as well as the effector cells in both CD4 and CD8. After antigen experience, central and effector memory T cells gain expression of *PTPRcv2* (CD45RO) and lose expression of CD45RA (43). The *TAGAP* encodes the T-cell activation Rho-GTPase-activating protein, and expression of *TAGAP* induced during T-cell activation (44). The function of TAGAP is currently unknown; however, a very recent study from del Rosario RC et al. (45 *)*, have reported that, in response to *Mtb* infection, an up-regulation of *TAGAP* involved in the enrichment of differential acetylation (DA) peaks in granulocytes. The nucleotide-binding domain, leucine-rich repeat-containing protein (NLR) family play key roles in innate immune defense, including protection against several major respiratory pathogens as well as in producing key Th1 and Th17 cell-promoting cytokines (46).

AERAS-402 incorporates Ad35, which has been shown to be prevalent in only 20% of individuals in sub-Saharan Africa (47) with neutralizing titers > 200 in less than 5% of individuals; this vector is able to induce potent immune responses against the encoded target antigen. Anti-Ad35–neutralizing antibodies were present in 25% of participants in this trial before AERAS-402 vaccination, which is noticeably higher than reported elsewhere (22). AERAS-402 post-vaccination at day 182, anti-Ad35–neutralizing antibodies were detected in half of the participants in this study. In contrast, anti-Ad35–neutralizing antibodies were detected a higher proportion in the placebo than AERAS-402 recipients.

To our knowledge, we are the first to explore transcriptional profiling in a phase 1 TB clinical trial and propose that immune related transcriptional biomarkers correlate with AERAS-402 recipients and the altered gene expression profiles are indicative of activation of immunologically relevant biological pathways. No safety concerns were observed for AERAS-402 in healthy Indian adult males. The vaccine also induced promising immunogenicity, predominantly to the Ag85B antigen consisting of polyfunctional T cell responses most robust for CD8 subsets, humoral immunity and altered gene expression profiles in PBMCs indicative of a localized activation of different biological pathways. Further, pre-existing antibodies towards the viral vector may likely impact on vaccine efficacy. Nonetheless, research on vaccine biomarkers has so far received little attention as an independent scientific priority from most of the main research-funding agencies and policymakers. More efforts are necessary to highlight the importance of vaccine biomarkers on the global vaccine agenda.

## Data Availability Statement

The raw data supporting the conclusions of this article will be made available by the authors, without undue reservation.

## Ethics Statement

The study was reviewed and approved by the Government of India Directorate General of Health Services, Office of the Drugs Controller General (Biological Division), Ref no: LL/RA/825/2007 and the Independent Ethics Committee Consultants Bangalore. The phase 1 clinical trial was registered at (https://clinicaltrials.gov/), and the identifier no: NCT01378312. Written informed consent was obtained from each participant prior to the conduct of any protocol-specific activity or study entry. The study was carried out following the ethical principles outlined in the Declaration of Helsinki and in accordance with the US Code of Federal Regulations for protection of human subjects (21 CFR 50), Institutional Review Boards (21 CFR 56), and the obligations of clinical investigators (21 CFR 312). The patients/participants provided their written informed consent to participate in this study.

## Author Contributions

DH performed the ICS. GB prepared the first draft; DH wrote sections on ICS and RB wrote the sections on transcription profiling together with DS and SJ. DS conducted the RNA extraction, dcRT-MLPA experiments. DS and CR performed the data analysis and generated **Table 5** and **Figures 1**, **4** and **5**, with contribution from SJ and HG. DS undertook revisions of manuscript with contribution from GB, DH, RB, CR, SJ, and HG. All authors contributed to the article and approved the submitted version.

## Funding

Research Council of Norway Global Health and Vaccination Research (GLOBVAC) projects: RCN 179342, 192534 and 248042, the University of Bergen (Norway); EDCTP2 programme supported by the European Union; the St. John’s Research Institute, Bangalore, India. Aeras funded by the Bill and Melinda Gates Foundation, Seattle WA.

## Conflict of Interest

The authors declare that the research was conducted in the absence of any commercial or financial relationships that could be construed as a potential conflict of interest.

## Glossary

Ag85: Antigen 85
*Ad*: Adenovirus
AE: adverse event
BCG: Bacillus Calmette-Guérin
*BD*: Becton Dickinson
*BMP*: Bone Morphogenetic Protein
*CD*: Cluster of differentiation 4
*CFR*: Code of Federal Regulations
*CCL*: C-C Motif Chemokine Ligand
CXCL: C-X-C motif chemokine
*CLEC*: C-Type Lectin domain Containing
*DCRT-MLPA*: Dual color Reverse Transcriptase-Multiplex Ligation Dependent Probe Amplification
*DMEM*: Dulbecco’s Modified Eagle Medium
DMSO: Dimethyl Sulfoxide
ELISA: Enzyme Linked Immunosorbent Assay
FunRich: Functional Enrichment Analysis
HIV: Human immunodeficiency virus
*GAPDH*: Glyceraldehyde-3-Phosphate Dehydrogenase
*GNLY*: Granulysin
*GUSB*: Glucuronidase Beta
*GZMA*: Granzyme A
IBM: International Business Machines
ICS: Intracellular Cytokine Staining
*IFN-γ*: Interferon gamma
*IFI*: Interferon Induced
*IFIT*: Interferon Induced protein with tetratricopeptide
*IL1B*: Interleukin 1 beta
*IL-2*: Interleukin 2
*KIF*: Kinesin Family Member
*LLOQ*: Lower limit of quantitation
Mtb: *M. tuberculosis*
*NLRP*: Nucleotide-binding oligomerization domain, Leucine rich Repeat and Pyrin domain containing
*NOD*: Nucleotide Binding Oligomerization Domain
PBMC: Peripheral blood mononuclear cells
*PCR*: Polymerase chain reaction
*PPD*: Purified protein derivative
*PTPRCv1*: Protein tyrosine phosphatase receptor type
QFT: QuantiFERON-TB Gold in-tube test
*RPMI*: Roswell Park Memorial Institute
SAE: Serious adverse events
*SE*: Standard Error
SEB: Staphylococcal enterotoxin B
WebGestalt: WEB-based GEne SeT AnaLysis Toolkit
*VEGF*: Vascular Endothelial Growth Factor
*TAGAP*: T Cell Activation Rho-GTPase Activating Protein
TB: Tuberculosis
*TLR*: Toll-like receptor
*TNF-α*: Tumor Necrosis Factor alpha
TST: Tuberculin Skin Test
*ZNF*: Zinc Finger Protein
