# Supplementary material for: A 2-Dose AERAS-402 Regimen Boosts CD8+ Polyfunctionality in HIV-Negative, BCG-Vaccinated Recipients
Source: Front Immunol. 2021 Jun 11;12:673532. doi: 10.3389/fimmu.2021.673532 (PMC8231292; doi:10.3389/fimmu.2021.673532)
Supplement: Supplementary file 1 [file Presentation_1.pptx]

## Slide 1
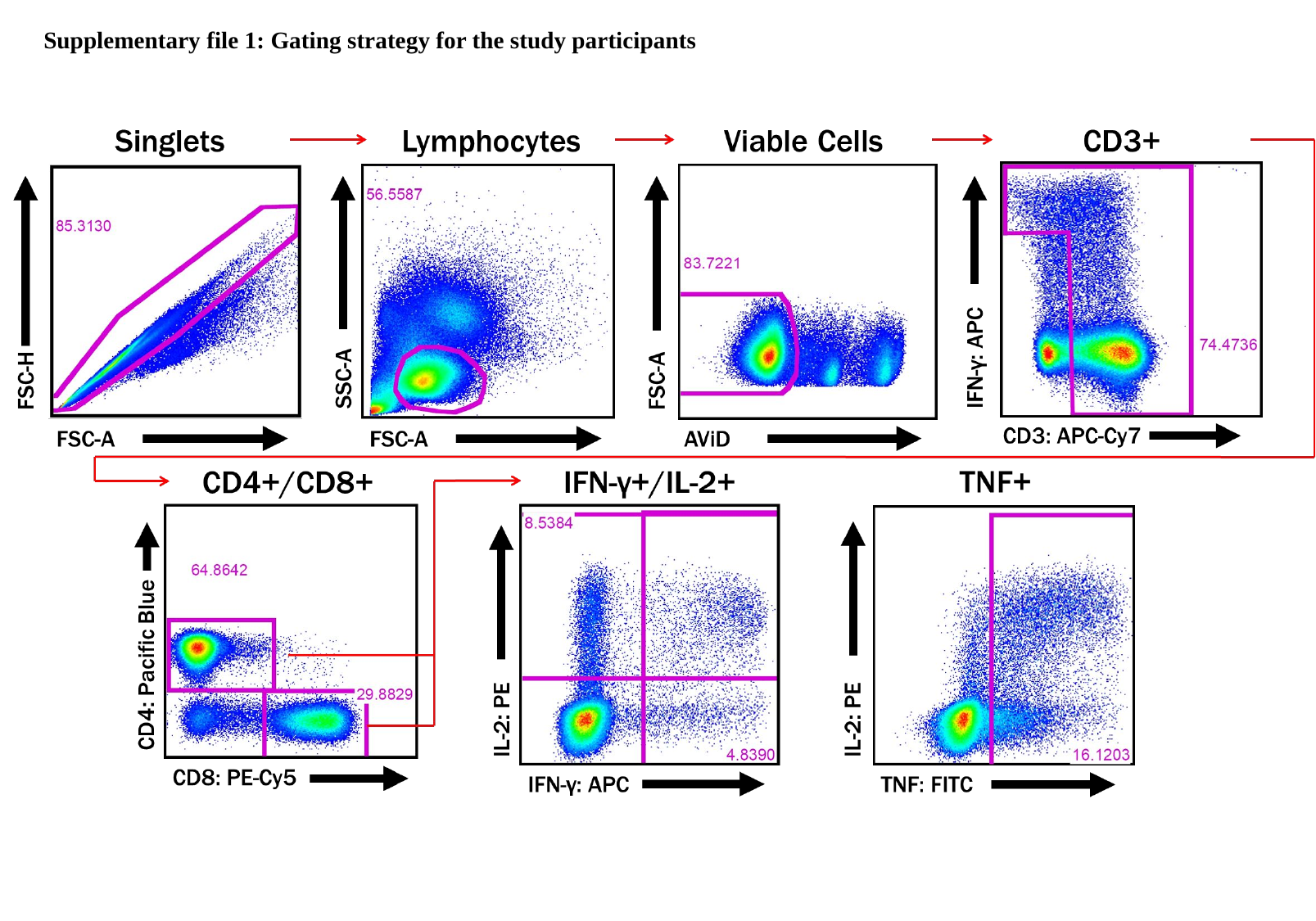

Supplementary file 1: Gating strategy for the study participants

## Slide 2
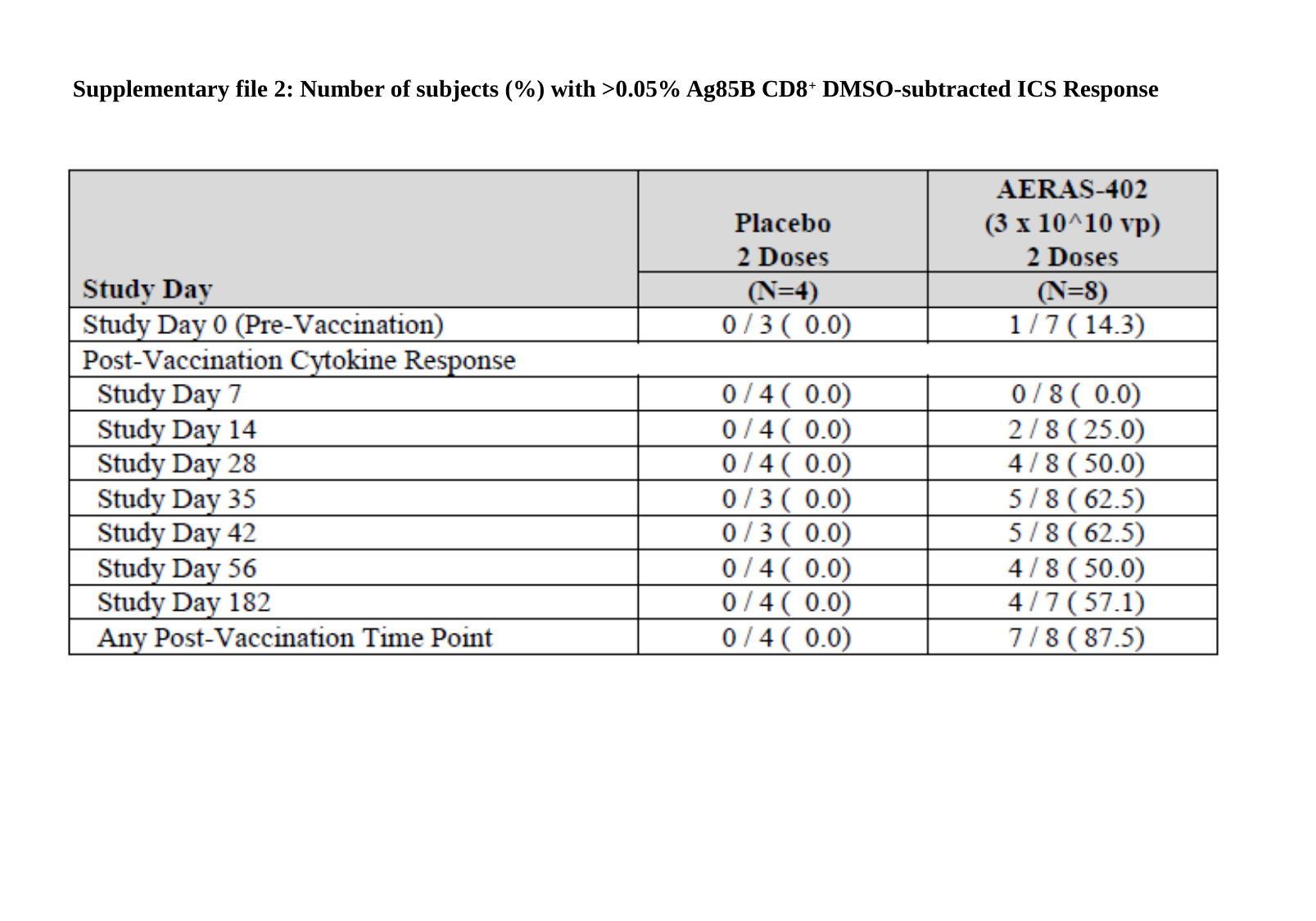

Supplementary file 2: Number of subjects (%) with >0.05% Ag85B CD8+ DMSO-subtracted ICS Response

## Slide 3
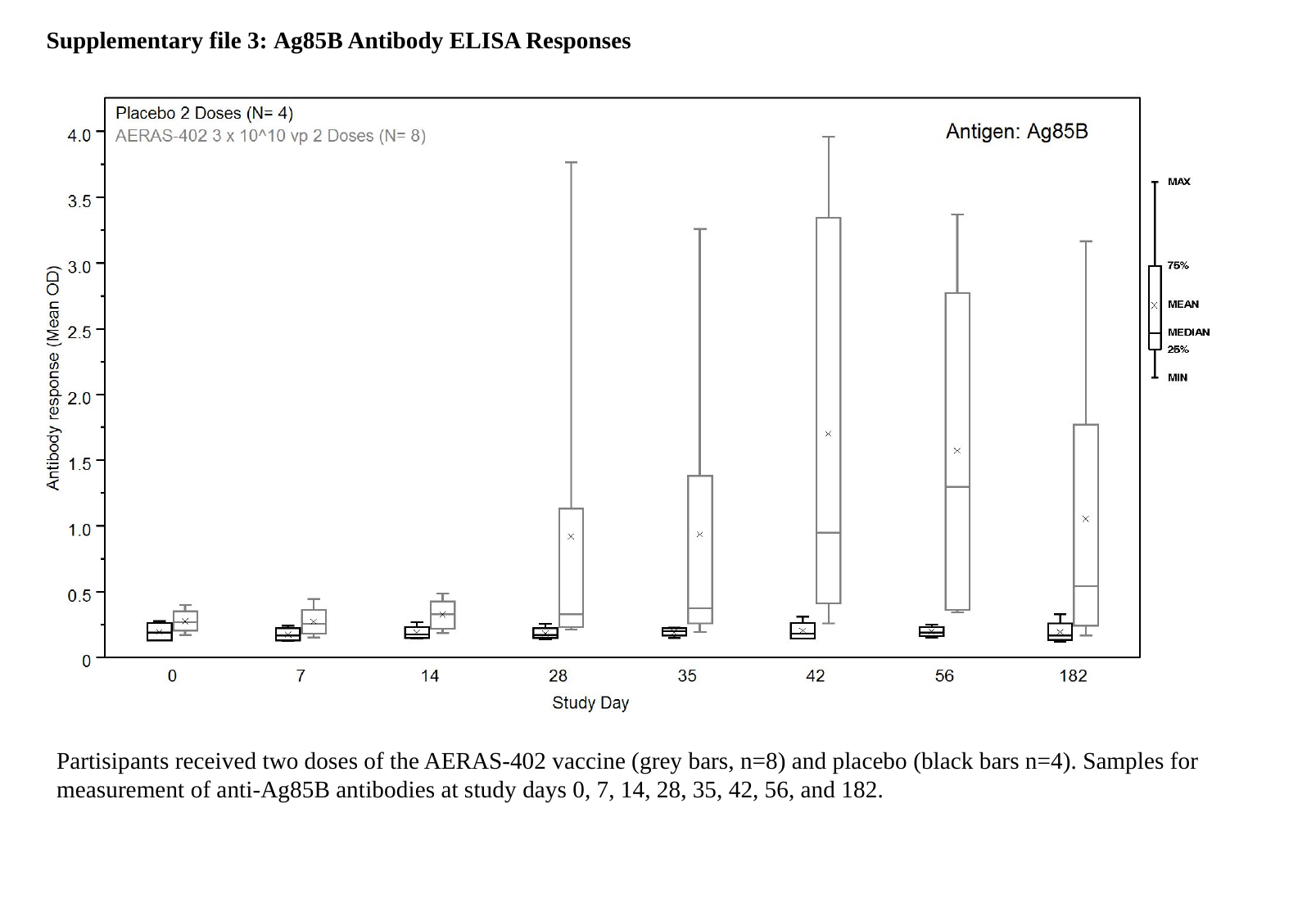

Supplementary file 3: Ag85B Antibody ELISA Responses
Partisipants received two doses of the AERAS-402 vaccine (grey bars, n=8) and placebo (black bars n=4). Samples for measurement of anti-Ag85B antibodies at study days 0, 7, 14, 28, 35, 42, 56, and 182.
